# Which statistical significance test best detects oncomiRNAs in cancer tissues? An exploratory analysis

**DOI:** 10.18632/oncotarget.12828

**Published:** 2016-10-23

**Authors:** Wei Tang, Zhijun Liao, Quan Zou

**Affiliations:** ^1^ Department of Biological Engineering, School of Chemical Engineering, Tianjin University, Tianjin, China; ^2^ Department of Biochemistry and Molecular Biology, School of Basic Medical Sciences, Fujian Medical University, Fuzhou, China; ^3^ School of Computer Science and Technology, Tianjin University, Tianjin, China; ^4^ State Key Laboratory of Medicinal Chemical Biology, Nankai University, Tianjin, China

**Keywords:** microRNA, differential expression, statistical significance test, MARS, oncomiRNA

## Abstract

MicroRNAs(miRNAs) often exert their oncogenic and tumor suppressor functions by suppressing protein-coding genes expressions in cancers and thus have a strong association with cancers' generation, development and metastasis. Through comprehensively understanding differentially expressed miRNAs (oncomiRNA) in tumor tissues, we can elucidate the underlying molecular mechanisms in tumorigenesis and develop novel strategies for cancer diagnosis and treatment. The differential expression of miRNAs can now be analyzed through numerous statistical significance tests based on different principles, which are also available in various R packages. However, the results can be notably different. In this study, we compared miRNAs obtained from 6 common significance tests/R packages (t-test, Limma, DESeq, edgeR, LRT and MARS) with the miRNAs archived in two databases; HMDD 2.0 database, which collects experimentally validated differentially expressed miRNAs, and Infer microRNA-disease association database*,* which contains the potential disease-associated miRNAs by network forecasting. Finally, we sought the MARS method in DEGseq package more effectively searched out differentially expressed miRNAs than other common methods.

## INTRODUCTION

MicroRNAs (miRNAs) are short (18-25-nucleotide) non-coding RNAs that function as posttranscriptional gene regulators by binding to the 3’UTR of mRNAs, consequently, either repress translation or initiate mRNA degradation [1, 2]. Since their discovery [3, 4], miRNAs have been implicated in the control of various cellular processes [5, 6], including cell proliferation [1], cell death [7-12] and differentiation [3, 13]. Therefore, many miRNAs could function as oncogenic miRNAs (oncomiRNAs), which cause cancer by down-regulating genes through both translational repression and mRNA destabilization mechanisms [14, 15], such as breast tumors [16, 17], esophageal carcinoma [18, 19] and lung cancer [1, 3]. miRNAs are also potential prognostic markers of chronic lymphocytic leukemia [20], colon tumors [15, 21], pancreatic cancer [22], and neuroblastoma [23]. Associations between differentially expressed (DE) miRNAs and the cancer occurrence have been the focus of intense cancer biology investigation [24-28].

Next Generation Sequencing (NGS) technology can rapidly and accurately perform large-scale DNA/RNA sequencing through a series of high-throughput technologies. These technologies facilitate genomic research and are increasingly replacing microarrays with gene expressing profiling of epigenetics and transcriptomics (RNA-seq) [29, 30]. Transcriptomic sequencing includes mRNA, small RNA and non-coding RNA (ncRNA), of which miRNAs are among the most important components [31-33]. Aided by the advantages of NGS, molecular biology has acquired a vast number of large-scale sequence data, which has also posed many challenges for high-throughput analysis. These challenges include finding suitable statistical tests for large data and affirming their statistical assumptions by biological experiments, such as quantitative RT-PCR, northern blot, and overcoming the shortcomings of genetic sequencing technologies through statistical methods, which should fully uncover the essence of biology. Selective miRNAs expression profiling based on high-throughput test can strongly support the prognosis prediction of various cancers [34, 35]. Therefore, a significance test or R package which can efficiently screen out DE miRNAs in tumor tissues will guide the subsequent validation by low-throughput experiments.

Various normalizations and statistical hypotheses have been incorporated into statistical significance tests and R packages which can detect DE miRNAs in cancer tissues. For example, the t-test (Student's t-test) is widely used for comparing independent samples by statistical hypothesis test. This test examines whether the expressions of certain miRNAs significantly differ among different parent population samples. A 2011 study compared the miRNAs expressions in 20 patients with glioblastoma and other 20 age- and sex-matched healthy controls [11]. The researchers identified 52 significant DE miRNAs among 1158 tested miRNAs in glioblastoma tissues, however, only 2 miRNAs (miR-128 and miR-342-3p, which are up- and down-regulated respectively) of these 52 miRNAs were validated by low-throughput real-time PCR experiments, which means only two miRNAs were suitable biomarkers for blood-derived glioblastoma-associated characteristic miRNA fingerprints [36]. The Limma package analyses gene expression data obtained from microarrays or RNA-seq technologies. The core capability of this package is the evaluation of differential expression in multifactor-designed experiments by linear modeling [37]. Sun [38] used the Limma package to screen out numerous DE miRNAs in ductal carcinoma in situ compared with normal controls . The DESeq [39] and edgeR [40] package solve the overdispersion problem in RNA sequencing data by applying the negative binomial distribution. Hamfjord [41] used both tools to statistically test the miRNA expression differences in read counts per miRNA between two samples. In DESeq, they treated the tumor and normal samples as independent groups; in edgeR, they considered paired information. According to their results, 37 miRNAs were identified to be DE miRNAs (19 up-regulated and 18 down-regulated) in colorectal cancer both in DESeq and edgeR [41], however, among these miRNAs, 16 miRNAs had not been validated in previously documented experiments. Thus, DESeq and edgeR both have limited screening ability for DE miRNAs. In 2010, Wang [42] proposed MA-plot-based method with random sampling (MARS) in DEGseq package. This method incorporates the random sampling method and is based on MA plot, which is widely used to detect and visualize the intensity-dependent ratios in microarray data [43]. DEGseq package also includes another commonly used method called Likelihood Ratio Test (LRT) [44].

Despite the wide range of statistical significance tests and R packages for detecting DE miRNAs in RNA expression profiles, few studies have considered which method gives the most accurate result. Along with the flourishing development of bioinformatics and applications of machine learning [24, 26, 45, 46], literature mining [47, 48] has greatly assisted biomedicine and genomics research. The HMDD 2.0 database (http://www.cuilab.cn/hmdd) collects experimental evidences of DE miRNAs and disease associations through literature mining [47]. The Infer microRNA-disease association database (http://lab.malab.cn/soft/ifmda) [49] predicts the underlying interactions between miRNAs and disease for further confirmation of biological experiments. The Infer method constructs a heterogeneous network that connects the disease similarity subnetwork to the miRNA similarity subnetwork by validated experimental miRNA-disease associations. The HMDD 2.0 database contains the experimentally validated DE miRNAs, and the Infer microRNA-disease association database offers miRNAs that are potentially associated with diseases.

In this study, we selected 5 miRNA expression profiles of cancers (BRCA, ESCA, LUAD, PAAD and THCA), and their corresponding controls miRNA profiles. We then screened the DE miRNAs in the cancer tissues by the six abovementioned methods (t-test, Limma, DESeq, edgeR, LRT and MARS), then compared and classified the six sets of results with the miRNAs in HMDD 2.0 and Infer microRNA-disease association. By calculating the Area Under the Curve (AUC) of the Receiver Operating Characteristic (ROC), we identified the method with the best screening results. Among the six methods, MARS delivered the highest performance.

## RESULTS

### Expression analysis of miRNAs in cancer *vs* normal groups

**Figure 1:**
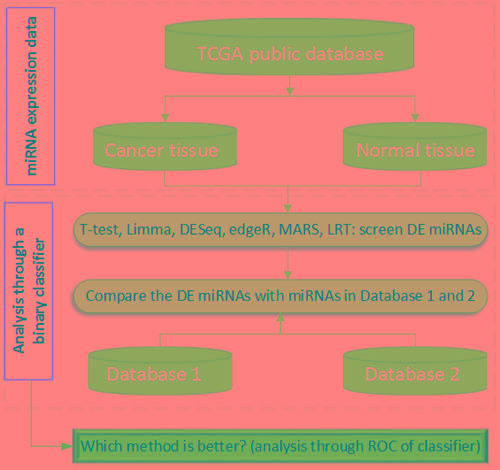
The flowchart to analyze miRNA and to compare with the other two databases in this study Note: DE: differentially expressed. Database 1: HMDD 2.0 database. Database 2: Infer microRNA-disease association database

**Figure 2:**
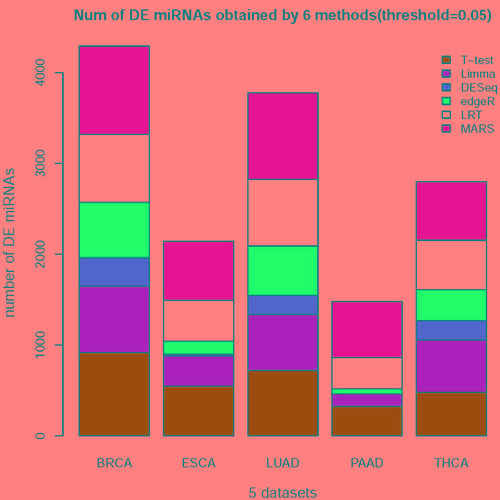
The histogram of the number of DE miRNAs obtained from 6 methods on 5 datasets The DE miRNAs were obtained from 6 methods(*t*-test, Limma, DESeq, edgeR, LRT and MARS) and the threshold: *P* or *P* < 0.05

After applying the six methods to the five datasets (Figure 1 and Table 1), we acquired the significant DE miRNAs in cancer tissues (*P* or *P_adj_* < 0.05) among the miRNAs. The numbers of DE miRNAs returned by the six methods have huge differences (Figure 2).

**Table 1.1:** Number of samples of selected miRNA sequencing datasets from the TCGA database

Cancers	NT samples	TN samples
BRCA	90	90
ESCA	13	13
LUAD	45	45
PAAD	4	4
THCA	57	57

**Table 1.2:** The number of selected miRNA from the database 1 and database 2

Cancers	TCGA	Database 1	Database 2
BRCA	1881	243	100
ESCA	1881	83	100
LUAD	1881	157	100
PAAD	1881	117	100
THCA	1881	56	100

Note:

NT: normal sample; TN: tumor sample;

Database 1: HMDD 2.0; Database 2: Infer microRNA-disease association;

BRCA: Breast invasive carcinoma

ESCA: Esophageal carcinoma

LUAD: Lung adenocarcinoma

PAAD: Pancreatic adenocarcinoma

THCA: Thyroid carcinoma.

**Figure 3:**
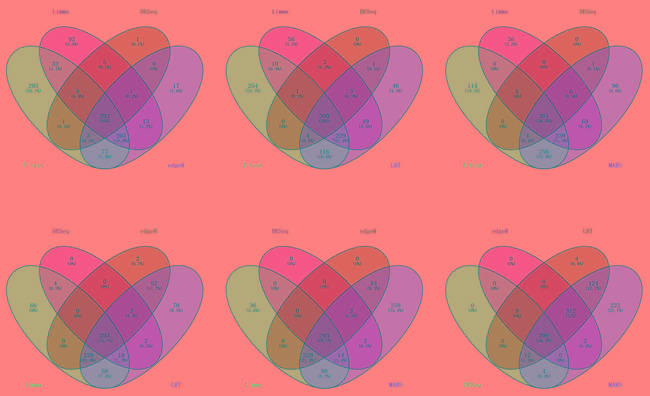
The venn diagrams of BRCA dataset BRCA dataset: The DE miRNAs were obtained from 6 methods(*t*-test, Limma, DESeq, edgeR, LRT and MARS) and the threshold *P* or *P*< 0.05, since venn diagrams based on 6 sets looks not intuitionistic, so we choose every 4 sets in 6 sets to draw venn diagrams.

The DE miRNAs in BRCA obtained by those 6 methods were compared in a Venny distribution using the Venny web server 2.0 (http://bioinfogp.cnb.csic.es/tools/venny/index.html) [50] (Figure 3). The miRNAs obtained from the six methods were also very different. For example, in BRCA dataset, hsa-mir-4482 was classified as a DE miRNA by Limma and edgeR, but as a normally expressed miRNA in DESeq.

These two figures indicated the large differences on the DE miRNAs obtained from those 6 methods. The differences not only lie in the number of those miRNAs but also in the predicted DE miRNAs.

### Compared with the miRNAs in HMDD 2.0 and Infer microRNA-disease association

**Figure 4:**
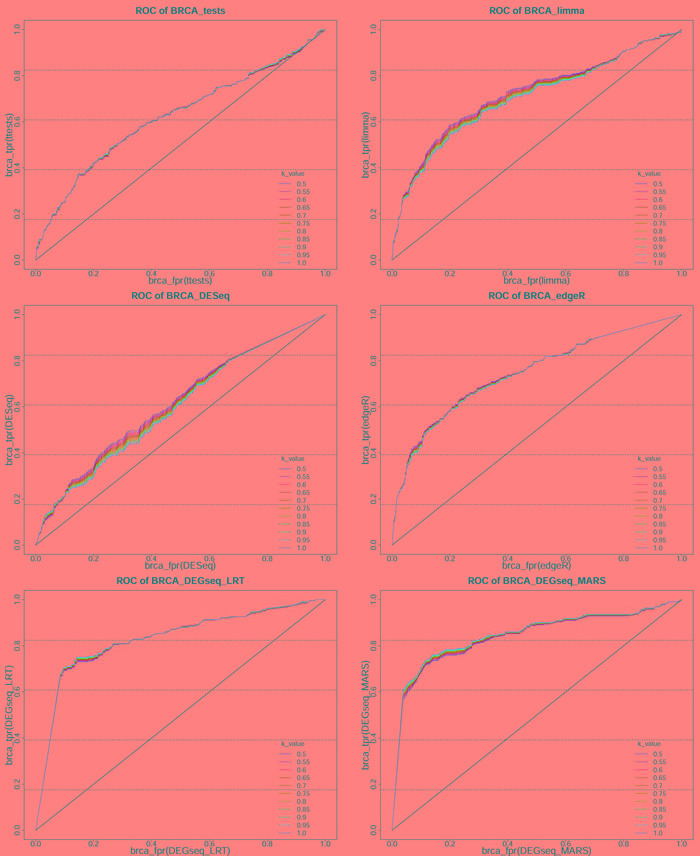
The ROC of 6 methods on BRCA dataset based on integrated HMDD 2.0 and Infer microRNA-disease association These ROC are obtained from classification of miRNAs obtained from 6 methods (*t*-test, Limma, DESeq, edgeR, LRT and MARS) on 5 datasets based on the true class in integrated HMDD 2.0 and Infer microRNA-disease association and *k*-value is the weighting coefficient, which is arithmetic progression from 0.5-1 with the step size equaling to 0.05

The total number of DE miRNAs can be varied by adjusting the threshold (*P* or *P_adj_*) of the six methods. Here, we classified the miRNAs obtained by the six methods with miRNAs in the HMDD 2.0 and Infer microRNA-disease association database. As these classifications are binary classifications (Table 2), we can estimate the screening DE miRNA performance of these methods by plotting the ROCs and computing their AUCs. When integrating the miRNAs from HMDD 2.0 and Infer microRNA-disease association, we incremented the *k*-value from 0.5 to 1 in 0.05 steps as a weight factor (Figure 4, Figures S1-S4; Table S1). When individually considering these two databases, we constructed separate ROCs for the miRNA comparisons between each statistical method and HMDD 2.0, and between each method and Infer microRNA-disease association (Figrue 5, Figures S5-S8; Table S2).

**Table 2:** The binary classifier

	True class	
Hypothesized class	*TP*(*True Positives*)	*FP*(*False Positives*)
	*FN*(*False Negatives*)	*TN*(*True Negatives*)

Note:

*TP*: true positive, the number of predicted miRNAs by statistical methods and also that appear in HMDD 2.0 or Infer microRNA-disease association

*FP*: false positive, the number of predicted miRNAs by statistical methods but not appear in HMDD 2.0 and Infer microRNA-disease association

*TN*: true negative, the number of not predicted miRNAs by statistical methods and also not appear in HMDD 2.0 and Infer microRNA-disease association

*FN*: false negative, the number of not predicted miRNAs by statistical methods but still appear in HMDD 2.0 or Infer microRNA-disease association

*Hypothesized class*: the predicted miRNA by those 6 methods

*True class*: the miRNA achieved in those 2 databases, HMDD 2.0 and Infer microRNA-disease association

**Figure 5:**
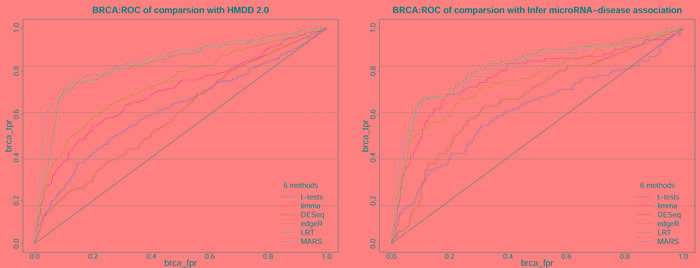
The ROC of 6 methods on BRCA dataset based on independently HMDD 2.0 and Infer microRNA-disease association These ROC are obtained from classification of miRNAs obtained from 6 methods (*t*-test, Limma, DESeq, edgeR, LRT and MARS) on 5 datasets based on the true class in independently HMDD 2.0 and Infer microRNA-disease association.

## DISCUSSION

Abnormal mRNA expression are induced by DE miRNAs, which prevents the mRNA from executing its regular biological functions [51-53], which is a primary cause of cancer. Altered miRNA expression will likely contribute to the initiation and progression of human cancers [10, 11, 13, 14, 16, 47], and the relationship between miRNAs and cancers has become a major focus in cancer research. Vast numbers of miRNA expression profiles have been generated throughout the past decade, as rapid NGS development has continuously lowered the gene sequencing cost. Although DE miRNAs in tumor tissues can be detected by various available methods, the accuracy of these methods remains a critical issue.

DE genes have been ubiquitously detected by the t-test, which is popular for its simple calculation and easily understandable characteristics. Even though the standard error in the t-test is based on a small sample size, some miRNAs with miniscule standard error will still inevitably exist among the great number of miRNAs. Consequently, the t-test will increase the false positives prediction for these miRNAs [54, 55]. Examining the ROC of PAAD dataset which has only 4 cancer samples and 4 control samples, we could observe clearly that the t-test cannot selectively screen the DE miRNAs in this dataset (Figure S3, Figure S7). In addition, the performance of t-test method on PAAD dataset was worse than any other methods when we compare the results with both integrated and independent considerations of HMDD 2.0 and Infer microRNA-disease association.

To improve the estimates stability in the traditional t-test, Limma introduces a prior distribution which can strengthen the sample variance estimation. The results of ROCs clearly showed that Limma delivered much better performance than the t-test in the small sample case, such as ESCA and PAAD datasets (Figure S1, Figure S3, Figure S5, Figure S7; Table S2). Limma also outperformed the t-test in the remaining datasets.

The DESeq and edgeR packages are based on the negative binomial (NB) distribution. The NB model corrects the overdispersion problem in RNA sequence data by an additional term in the variance of the Poisson model. The variance parameter is estimated differently in DESeq and edgeR; DESeq estimates the mean-dependent dispersion by a local regression method, whereas edgeR assumes that the mean and variance are related and thus share a single common estimate of the dispersion parameter across the read counts. edgeR also weakens each miRNA's the dispersion degree through an empirical Bayes method [40]. We note that many statistical methods cannot properly handle the small sample sizes which are very common in RNA sequencing experiments, for example, in DESeq, which are based on generalized linear models, small sample sizes consequently violated the assumptions of its statistical tests [39, 40]. As a result, DESeq was almost the worst performer in our experiments (Figures S1-S8). The edgeR method also inflated the type I error rates in the simulations. The results of both DESeq and edgeR deviated largely in relatively small samples, such as the PAAD (4) and ESCA (13) dataset (Table 1.1). The AUCs of both methods were also quite different (Tables S1-S2); in the ESCA dataset, the AUC in edgeR was 0.18-0.2 larger than in DESeq, and in the PAAD dataset, the DESeq was even ineffective due to the very small sample size. In other three datasets, which has relatively larger sample sizes than PAAD and ESCA, reduced the differences between performances in edgeR and DESeq (Tables S1-S2). Our simulations also confirmed a higher computational speed of edgeR than DESeq, moreover, the latter could even incur memory leakage at relatively large sample sizes.

According to the technical characteristic of RNA-Seq, Wang [42] proposed the MARS method in DEGseq, which can detects DE genes from MA plots and its test hypothesis is based on a random sampling model [42]. The DEGseq package includes LRT as well. Both methods were demonstrated higher DE miRNA screening performance in all five datasets than the other four methods (t-test, Limma, DESeq and edgeR). The results showed some AUCs of MARS and LRT were close to 0.90 and some were much higher than 0.9, suggesting that the both methods are very effective on detecting DE miRNAs (Figures S1-S8, Tables S1-S2). Moreover, the AUCs of the PAAD and ESCA dataset confirmed that MARS and LRT can also effectively identify DE miRNAs even in small samples. Although both MARS and LRT performed well, MARS achieved a higher True Positive Rate (TPR) at low False Positive Rate (FPR) than LRT in the BRCA, ESCA, LUAD and THCA datasets, in PAAD dataset, MARS and LRT achieved nearly identical TPR at the same low FPR (Figures S3 and S7), which indicated that MARS could correctly identify DE miRNAs with fewer misidentified miRNAs than LRT (Figures S1-S8). In summary, among the six tested methods, MARS could most accurately detecte the DE miRNAs in cancer tissues.

The detection of DE miRNAs in cancers (oncomiRNAs) has always been among the most important issue in cancer biology research. Prior accurate computational detection of DE miRNAs will effectively reduce the cost of clinical experiments. Current computational analyses focus on statistical significance tests [56], literature mining and networking prediction [57]. However, few works have considered all of these approaches. In the present study, we integrated these three approaches to maximize the uniformity of the results to see which method could most accurately detect DE miRNAs. The DE miRNAs detected by the best performer (MARS) were highly consistent with the miRNAs extracted from literature mining and network prediction. This supports our inference that MARS outperforms other statistical significance tests (such as t-tests) in DE miRNAs detection. Complex genetic regulatory mechanisms in high-level organisms is considered to be achieved through controlled and coordinated miRNAs networks. The associations between miRNAs and disease are not only conducive to develop novel therapeutic applications for cancer patients by miRNA delivery and inhibition, but also help to construct the RNA network which is crucial to understand the underlying mechanisms of genetic network. In future work, we will continue to focus on improving the accuracy of oncomiRNA detection through integrating significance tests, literature mining and network prediction with including more data resources and also involving machine learning methods modify the MARS method.

## MATERIALS AND METHODS

### Flowchart

Figure 1 is a flowchart of the present study. After a comprehensive analysis of miRNA expression in cancerous and normal tissue samples, the results were compared with the miRNAs in HMDD 2.0 and the Infer microRNA-disease associations. Finally, we identified the best DE miRNA detection method among the six statistical significance tests/R packages.

### Source data and sequence expression analysis

In this study, we selected five deep sequencing miRNA datasets from The Cancer Genome Atlas (TCGA) pilot project (https://tcga-data.nci.nih.gov/tcga/). All of these data were sequenced by the BCGSC (IlluminaHiSeq_miRNAseq) sequencing platform. HMDD 2.0 database collects DE miRNAs in various cancer types which were validated by biological experiments. However, many of the cancer types in HMDD 2.0 have not collected sufficiently experimentally validated miRNAs, only 5 cancer types not only collect more than 50 validated DE miRNAs in HMDD 2.0, included the miRNA sequences of breast invasive carcinoma (BRCA), esophageal carcinoma (ESCA), lung adenocarcinoma (LUAD), pancreatic adenocarcinoma (PAAD) and thyroid carcinoma (THCA), but also have corresponding miRNAs in Infer microRNA-disease association database. So in order to assure the statistical significance, we selected these 5 datasets (BRCA, ESCA, LUAD, PAAD and THCA) for further analysis. As the sample sizes differed between tumor and normal samples, we randomly selected corresponding tumor samples with the same and similar characteristics (Table 1).

After obtaining the miRNA expression profiles from TCGA (the original sequencing data had been subjected to mapping analysis), we analyzed the miRNAs through abovementioned statistical methods (t-test, Limma, DESeq, edgeR, LRT and MARS), and computed the corresponding *P* or *P_adj_* (the associated FDR (False Discovery Rate)) value of each miRNA in the five datasets. Results were deemed significant at the *P* or *P_adj_* = 0.05 level.

### Statistical comparison with HMDD 2.0 and Infer microRNA-disease association

If the *P* or *P_adj_* value was below 0.05, the miRNA expression between the cancer and normal samples was considered as statistically significant, and the miRNA was assumed as a DE miRNA in the tumor tissue. By varying *P* or *P_adj_*, we can vary the numbers of miRNAs that pass the hypothesis test.

All of the tested methods generated a *P* or *P_adj_* value for each miRNA in the miRNAs differential expression analysis. Therefore, the comparison between the miRNAs obtained from these six methods and those in HMDD 2.0 database and Infer microRNA-disease association database can be regarded as a binary classification process. Here, the predicted outputs are the miRNAs obtained from the significance tests/R packages, and the true classes are the miRNAs of the corresponding cancers in HMDD 2.0 and Infer microRNA-disease association. The matrix so constructed is the basis of many common metrics (Table 2)

The performance of a binary classification could be characterized by two basic measurements; the recall rate and precision rate. However, these measurements are of limited usefulness due to their single-valued feature [58], we instead computed the True Positive Rate (TPR) and False Positive Rate (FPR), then plotted the ROC, which could be quantified by the AUC. In biomedical applications, the ROC is commonly used to judge the performance of a discriminant across varying decision thresholds [59, 60], and it has become increasingly important in the classification of unequally distributed categories, since its unique attributes can handle unequal costs incurred by classification errors [59, 60]. Thus, the ROC could provide a better metric than the accuracy measure in certain classifiers [61, 62]. In the binary classification of our DE miRNA analysis, we specified a threshold for the obtained outcome, such as 0.01, and assigned respectively all instances above and below this value as negative (no differential expression) and positive (differential expression). Increasing the threshold to 0.05 will increase the number of true positive instances, and thereby the proportion of true positives among all positive instances (the true positive rate, or TPR) increases. However, a higher threshold also classifies more negative instances as positive, increasing the false positive rate (FPR). The AUC of the ROC is another indicator of the classifier performance [63, 64]. The TPR and FPR are respectively calculated as:

$$TPR\left(TruePositiveRate\right)=\frac{TP}{TP+FN}$$ (1)

$$FPR\left(TruePositiveRate\right)=\frac{FP}{FP+TN}$$ (2)

Where True Positive (TP*)* and False Positive (FP) denote the numbers of positive and negative samples that are classified as positive, respectively, and True Negative (TN) and False Negative (FN) represent the corresponding values of the negative samples.

Because the AUC is a portion of a unit square area, its value always lies in the range 0-1.0. Another important statistical property of AUC is that the value equals the probability that the classifier will rank a randomly chosen positive instance higher than a randomly chosen negative instance. A higher AUC shifts the ROC toward the upper-left of the square, indicating higher performance of the classifier [60]. As the miRNAs in HMDD 2.0 were validated by biological experiments and those miRNAs in Infer microRNA-disease association were predicted by network, we must assign a weighting coefficient when considering the true classes in an integrated manner. Here, we applied a simple linear function. Specifically, we modified the TP and FN by introducing a parameter *k*:

$$TP\left(True\;Positive\right)=\frac{k*sum1}{\left(1-k\right)*sum2}$$ (3)

$$FN\left(False\;Positive\right)=\frac{k*\left(sum3-sum1\right)}{\left(1-k\right)*\left(sum3-sum2\right)}$$ (4)

In Eqs. (3) and (4), *sum*1 and *sum*2 are the sums of the miRNAs appearing in HMDD 2.0 (correctly classified positives in HMDD 2.0) and Infer microRNA-disease association (correctly classified positives in the Infer database), respectively, and *sum*3 is the total sum of the miRNAs obtained by all methods.

As the miRNAs obtained from Infer microRNA-disease association might not be associated with cancers, they should be weighted less heavily than those in HMDD 2.0, whose associations with cancer are confirmed. Hence, the *k*-value was varied as 0.5 ≤ *k ≤* 1

## SUPPLEMENTARY MATERIALS FIGURES AND TABLES





## References

[1] Bartel DP (2004). MicroRNAs: genomics, biogenesis, mechanism, and function. cell.

[2] Liu B, Fang L, Liu F, Wang X, Chen J, Chou K-C (2015). Identification of real microRNA precursors with a pseudo structure status composition approach. PLoS ONE.

[3] Lee RC, Feinbaum RL, Ambros V (1993). The C. elegans heterochronic gene lin-4 encodes small RNAs with antisense complementarity to lin-14. Cell.

[4] Wightman B, Ha I, Ruvkun G (1993). Posttranscriptional regulation of the heterochronic gene lin-14 by lin-4 mediates temporal pattern formation in C. elegans. Cell.

[5] Chen X, Yan CC, Zhang X, You Z-H (2016). Long non-coding RNAs and complex diseases: from experimental results to computational models. Briefings in Bioinformatics.

[6] Liu B, Fang L, Jie C, Liu F, Wang X (2015). miRNA-dis: microRNA precursor identification based on distance structure status pairs. Molecular BioSystems.

[7] Hatfield S, Shcherbata H, Fischer K, Nakahara K, Carthew R, Ruohola-Baker H (2005). Stem cell division is regulated by the microRNA pathway. Nature.

[8] Cheng L-C, Tavazoie M, Doetsch F (2005). Stem cells: from epigeneticsto microRNAs. Neuron.

[9] Houbaviy HB, Murray MF, Sharp PA (2003). Embryonic stem cell-specific MicroRNAs. Developmental cell.

[10] Suh M-R, Lee Y, Kim JY, Kim S-K, Moon S-H, Lee JY, Cha K-Y, Chung HM, Yoon HS, Moon SY (2004). Human embryonic stem cells express a unique set of microRNAs. Developmental biology.

[11] Zhang B, Pan X, Anderson TA (2006). MicroRNA: a new player in stem cells. Journal of cellular physiology.

[12] Wu D, Huang Y, Kang J, Li K, Bi X, Zhang T, Jin N, Hu Y, Tan P, Zhang L (2015). ncRDeathDB: A comprehensive bioinformatics resource for deciphering network organization of the ncRNA-mediated cell death system. Autophagy.

[13] Chen C-Z, Li L, Lodish HF, Bartel DP (2004). MicroRNAs modulate hematopoietic lineage differentiation. science.

[14] Lu J, Getz G, Miska EA, Alvarez-Saavedra E, Lamb J, Peck D, Sweet-Cordero A, Ebert BL, Mak RH, Ferrando AA (2005). MicroRNA expression profiles classify human cancers. nature.

[15] Volinia S, Calin GA, Liu C-G, Ambs S, Cimmino A, Petrocca F, Visone R, Iorio M, Roldo C, Ferracin M (2006). A microRNA expression signature of human solid tumors defines cancer gene targets. Proceedings of the National academy of Sciences of the United States of America.

[16] Yu F, Yao H, Zhu P, Zhang X, Pan Q, Gong C, Huang Y, Hu X, Su F, Lieberman J (2007). let-7 regulates self renewal and tumorigenicity of breast cancer cells. Cell.

[17] Iorio MV, Ferracin M, Liu C-G, Veronese A, Spizzo R, Sabbioni S, Magri E, Pedriali M, Fabbri M, Campiglio M (2005). MicroRNA gene expression deregulation in human breast cancer. Cancer research.

[18] Feber A, Xi L, Luketich JD, Pennathur A, Landreneau RJ, Wu M, Swanson SJ, Godfrey TE, Litle VR (2008). MicroRNA expression profiles of esophageal cancer. The Journal of thoracic and cardiovascular surgery.

[19] Kano M, Seki N, Kikkawa N, Fujimura L, Hoshino I, Akutsu Y, Chiyomaru T, Enokida H, Nakagawa M, Matsubara H (2010). miR-145, miR-133a and miR-133b: tumor-suppressive miRNAs target FSCN1 in esophageal squamous cell carcinoma. International Journal of Cancer.

[20] Calin GA, Liu C-G, Sevignani C, Ferracin M, Felli N, Dumitru CD, Shimizu M, Cimmino A, Zupo S, Dono M (2004). MicroRNA profiling reveals distinct signatures in B cell chronic lymphocytic leukemias. Proceedings of the National Academy of Sciences of the United States of America.

[21] Michael MZ, O'Connor SM, van Holst Pellekaan NG, Young GP, James RJ (2003). Reduced Accumulation of Specific MicroRNAs in Colorectal Neoplasia11Note: Susan M. O'Connor and Nicholas G. van Holst Pellekaan contributed equally to this work. Molecular Cancer Research.

[22] Bloomston M, Frankel WL, Petrocca F, Volinia S, Alder H, Hagan JP, Liu C-G, Bhatt D, Taccioli C, Croce CM (2007). MicroRNA expression patterns to differentiate pancreatic adenocarcinoma from normal pancreas and chronic pancreatitis. Jama.

[23] Schulte JH, Schowe B, Mestdagh P, Kaderali L, Kalaghatgi P, Schlierf S, Vermeulen J, Brockmeyer B, Pajtler K, Thor T (2010). Accurate prediction of neuroblastoma outcome based on miRNA expression profiles. International Journal of Cancer.

[24] Chen X, Clarence Yan C, Zhang X, You ZH, Huang YA, Yan GY (2016). HGIMDA: Heterogeneous graph inference for miRNA-disease association prediction. Oncotarget.

[25] Chen X, You ZH, Yan GY, Gong DW (2016). IRWRLDA: improved random walk with restart for lncRNA-disease association prediction. Oncotarget.

[26] Chen X, Yan G-Y (2014). Semi-supervised learning for potential human microRNA-disease associations inference. Scientific reports.

[27] Chen X, Huang YA, Wang XS, You ZH, Chan K (2016). FMLNCSIM: fuzzy measure-based lncRNA functional similarity calculation model. Oncotarget.

[28] Wang Y, Chen L, Chen B, Li X, Kang J, Fan K, Hu Y, Xu J, Yi L, Yang J (2013). Mammalian ncRNA-disease repository: a global view of ncRNA-mediated disease network. Cell Death & Disease.

[29] Morozova O, Marra MA (2008). Applications of next-generation sequencing technologies in functional genomics. Genomics.

[30] Metzker ML (2010). Sequencing technologies—the next generation. Nature reviews genetics.

[31] Keller C, Bühler M (2013). Chromatin-associated ncRNA activities. Chromosome Research.

[32] Mattick JS, Makunin IV (2006). Non-coding RNA. Human molecular genetics.

[33] Li Y, Wang C, Miao Z, Bi X, Wu D, Jin N, Wang L, Wu H, Qian K, Li C (2014). ViRBase: a resource for virus-host ncRNA-associated interactions. Nucleic Acids Research.

[34] Calin GA, Croce CM (2006). MicroRNA-cancer connection: the beginning of a new tale. Cancer research.

[35] Calin GA, Croce CM (2006). MicroRNA signatures in human cancers. Nature Reviews Cancer.

[36] Roth P, Wischhusen J, Happold C, Chandran PA, Hofer S, Eisele G, Weller M, Keller A (2011). A specific miRNA signature in the peripheral blood of glioblastoma patients. Journal of neurochemistry.

[37] Ritchie ME, Phipson B, Wu D, Hu Y, Law CW, Shi W, Smyth GK (2015). limma powers differential expression analyses for RNA-sequencing and microarray studies. Nucleic acids research.

[38] Sun E, Zhou Q, Liu K, Wei W, Wang C, Liu X, Lu C, Ma D (2014). Screening miRNAs related to different subtypes of breast cancer with miRNAs microarray. Eur Rev Med Pharmacol Sci.

[39] Anders S, Huber W (2010). Differential expression analysis for sequence count data. Genome biology.

[40] Robinson MD, McCarthy DJ, Smyth GK (2010). edgeR: a Bioconductor package for differential expression analysis of digital gene expression data. Bioinformatics.

[41] Hamfjord J, Stangeland AM, Hughes T, Skrede ML, Tveit KM, Ikdahl T, Kure EH (2012). Differential expression of miRNAs in colorectal cancer: comparison of paired tumor tissue and adjacent normal mucosa using high-throughput sequencing. PloS one.

[42] Wang L, Feng Z, Wang X, Wang X, Zhang X (2010). DEGseq: an R package for identifying differentially expressed genes from RNA-seq data. Bioinformatics.

[43] Yang YH, Dudoit S, Luu P, Lin DM, Peng V, Ngai J, Speed TP (2002). Normalization for cDNA microarray data: a robust composite method addressing single and multiple slide systematic variation. Nucleic acids research.

[44] Marioni JC, Mason CE, Mane SM, Stephens M, Gilad Y (2008). RNA-seq: an assessment of technical reproducibility and comparison with gene expression arrays. Genome research.

[45] Liu B, Zhang D, Xu R, Xu J, Wang X, Chen Q, Dong Q, Chou K-C (2014). Combining evolutionary information extracted from frequency profiles with sequence-based kernels for protein remote homology detection. Bioinformatics.

[46] Chen J, Wang X, Liu B (2016). iMiRNA-SSF: Improving the Identification of MicroRNA Precursors by Combining Negative Sets with Different Distributions. Scientific Reports.

[47] Li Y, Qiu C, Tu J, Geng B, Yang J, Jiang T, Cui Q (2013). HMDD v2. 0: a database for experimentally supported human microRNA and disease associations. Nucleic acids research.

[48] Jiang Q, Wang Y, Hao Y, Juan L, Teng M, Zhang X, Li M, Wang G, Liu Y (2009). miR2Disease: a manually curated database for microRNA deregulation in human disease. Nucleic Acids Research.

[49] Liu Y, Zeng X, He Z, Zou Q (2016). Inferring microRNA-disease associations by random walk on a heterogeneous network with multiple data sources. IEEE/ACM Transactions on Computational Biology and Bioinformatics.

[50] Oliveros JC (2007). VENNY. An interactive tool for comparing lists with Venn Diagrams.

[51] Valencia-Sanchez MA, Liu J, Hannon GJ, Parker R (2006). Control of translation and mRNA degradation by miRNAs and siRNAs. Genes & development.

[52] Guo H, Ingolia NT, Weissman JS, Bartel DP (2010). Mammalian microRNAs predominantly act to decrease target mRNA levels. Nature.

[53] Zhang B, Pan X, Cobb GP, Anderson TA (2007). microRNAs as oncogenes and tumor suppressors. Developmental biology.

[54] Cui X, Churchill GA (2003). Statistical tests for differential expression in cDNA microarray experiments. Genome biology.

[55] Baldi P, Long AD (2001). A Bayesian framework for the analysis of microarray expression data: regularized t-test and statistical inferences of gene changes. Bioinformatics.

[56] Guo L, Yu J, Liang T, Zou Q (2016). miR-isomiRExp: a web-server for the analysis of expression of miRNA at the miRNA/isomiR levels. Scientific Reports.

[57] Zou Q, Li J, Hong Q, Lin Z, Shi H, Wu Y, Ju Y (2015). Prediction of microRNA-disease associations based on social network analysis methods. BioMed Research International.

[58] Hripcsak G, Rothschild AS (2005). Agreement, the f-measure, and reliability in information retrieval. Journal of the American Medical Informatics Association.

[59] Fawcett T (2006). An introduction to ROC analysis. Pattern recognition letters.

[60] Pencina MJ, D'Agostino RB, Vasan RS (2008). Evaluating the added predictive ability of a new marker: from area under the ROC curve to reclassification and beyond. Statistics in medicine.

[61] Wang P, Tang K, Weise T, Tsang E, Yao X (2014). Multiobjective genetic programming for maximizing ROC performance. Neurocomputing.

[62] Hanczar B, Hua J, Sima C, Weinstein J, Bittner M, Dougherty ER (2010). Small-sample precision of ROC-related estimates. Bioinformatics.

[63] Lobo JM, Jiménez-Valverde A, Real R (2008). AUC: a misleading measure of the performance of predictive distribution models. Global ecology and Biogeography.

[64] Tang H, Chen W, Lin H (2016). Identification of immunoglobulins using Chou's pseudo amino acid composition with feature selection technique. Mol Biosyst.

