# Supplementary material for: Which statistical significance test best detects oncomiRNAs in cancer tissues? An exploratory analysis
Source: Oncotarget. 2016 Oct 23;7(51):85613–23. doi: 10.18632/oncotarget.12828 (PMC5356763; doi:10.18632/oncotarget.12828)
Supplement: Supplementary file 1 [file oncotarget-07-85613-s001.pdf]

Which statistical significance test best detects oncomiRNAs in cancer tissues? An exploratory analysis

Supplementary Material

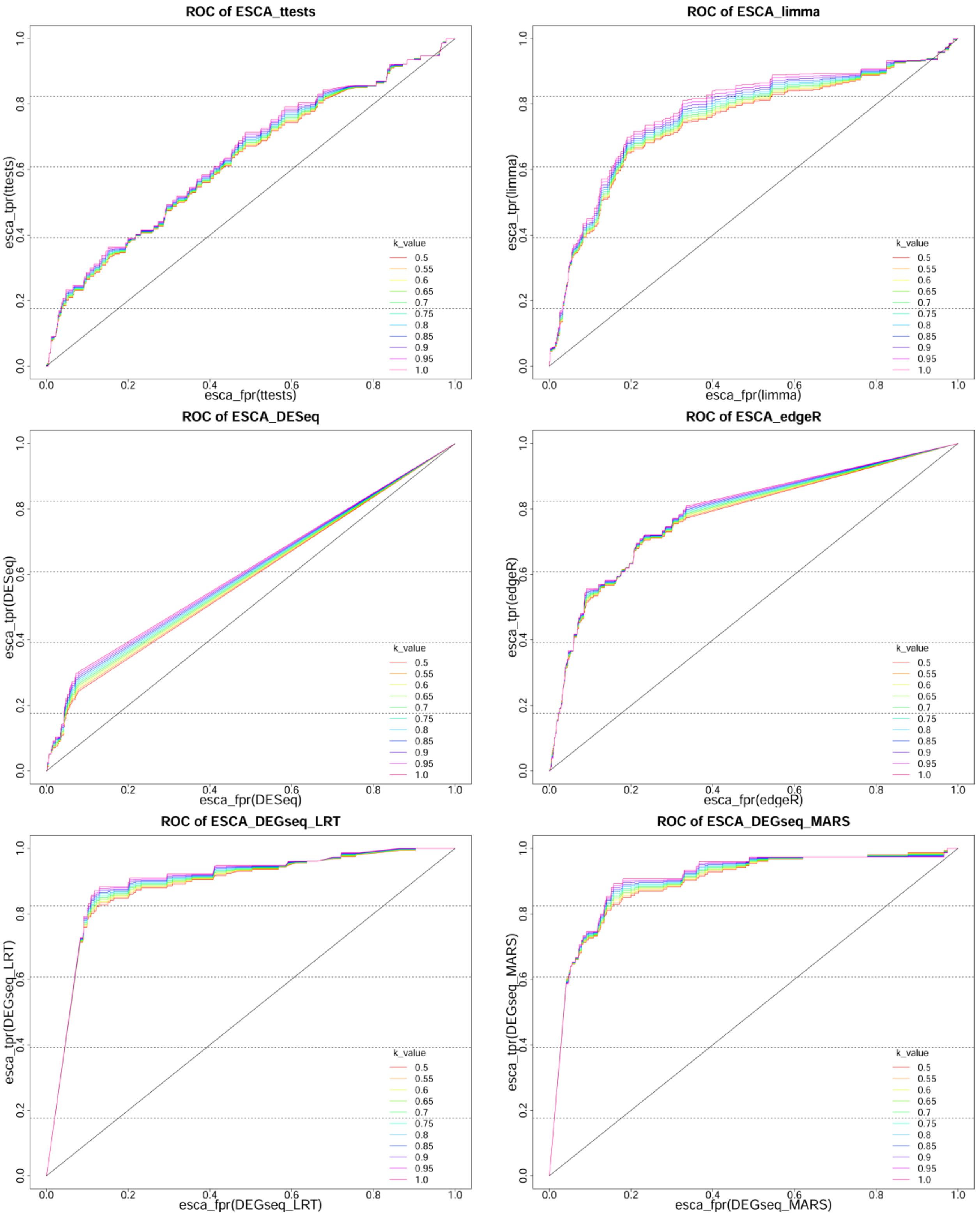
ROC of ESCA_ttests
esca_tpr(ttests)
esca_fpr(ttests)
ROC of ESCA_limma
esca_tpr(limma)
esca_fpr(limma)
ROC of ESCA_DESeq
esca_tpr(DESeq)
esca_fpr(DESeq)
ROC of ESCA_edgeR
esca_tpr(edgeR)
esca_fpr(edgeR)
ROC of ESCA_DEGseq_LRT
esca_tpr(DEGseq_LRT)
esca_fpr(DEGseq_LRT)
ROC of ESCA_DEGseq_MARS
esca_tpr(DEGseq_MARS)
esca_fpr(DEGseq_MARS)
k_value
0.5
0.55
0.6
0.65
0.7
0.75
0.8
0.85
0.9
0.95
1.0
0.0
0.2
0.4
0.6
0.8
1.0

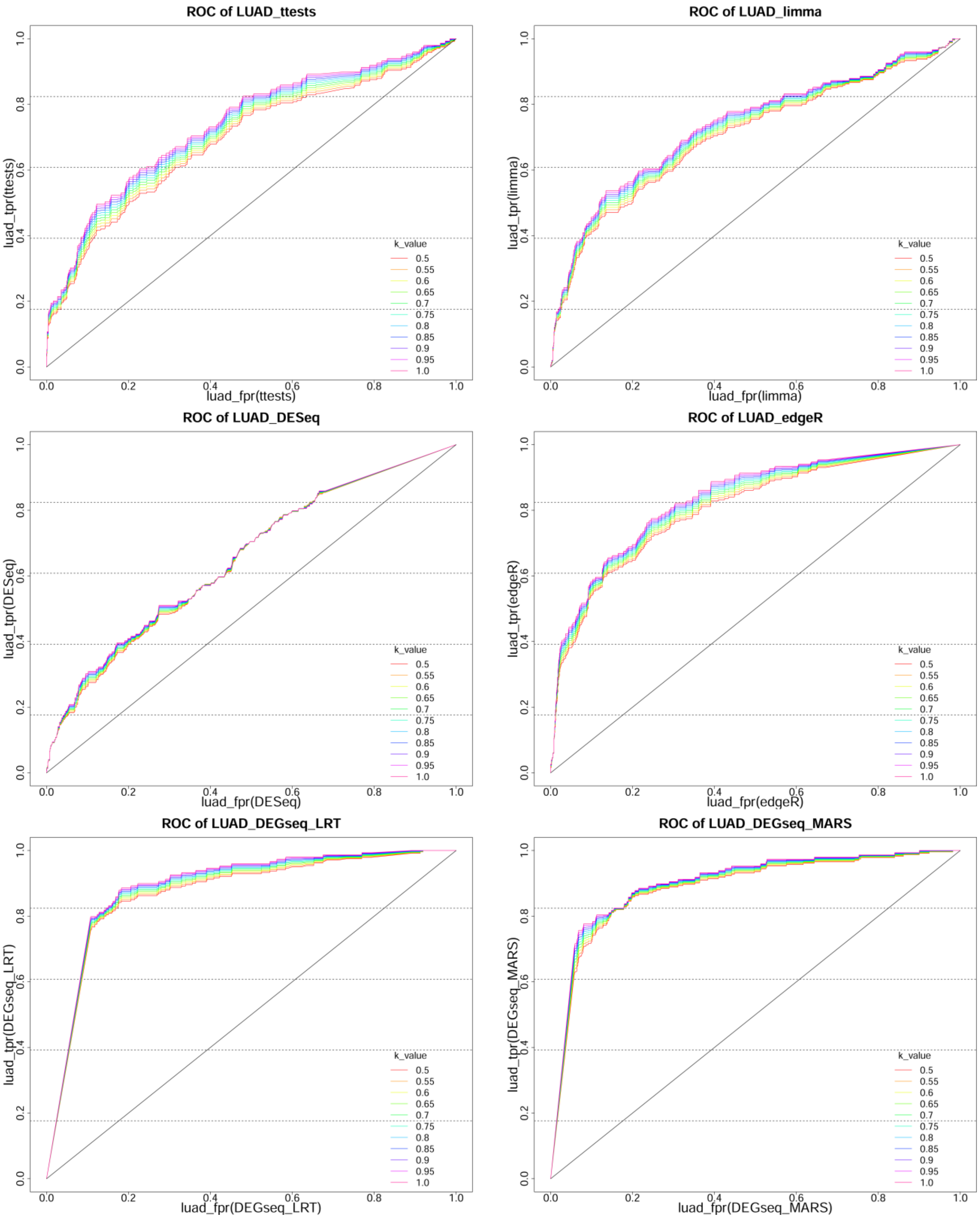
ROC of LUAD_ttests
luad_tpr(ttests)
luad_fpr(ttests)
ROC of LUAD_limma
luad_tpr(limma)
luad_fpr(limma)
ROC of LUAD_DESeq
luad_tpr(DESeq)
luad_fpr(DESeq)
ROC of LUAD_edgeR
luad_tpr(edgeR)
luad_fpr(edgeR)
ROC of LUAD_DEGseq_LRT
luad_tpr(DEGseq_LRT)
luad_fpr(DEGseq_LRT)
ROC of LUAD_DEGseq_MARS
luad_tpr(DEGseq_MARS)
luad_fpr(DEGseq_MARS)
0.0
0.2
0.4
0.6
0.8
1.0
k_value
0.5
0.55
0.6
0.65
0.7
0.75
0.8
0.85
0.9
0.95
1.0

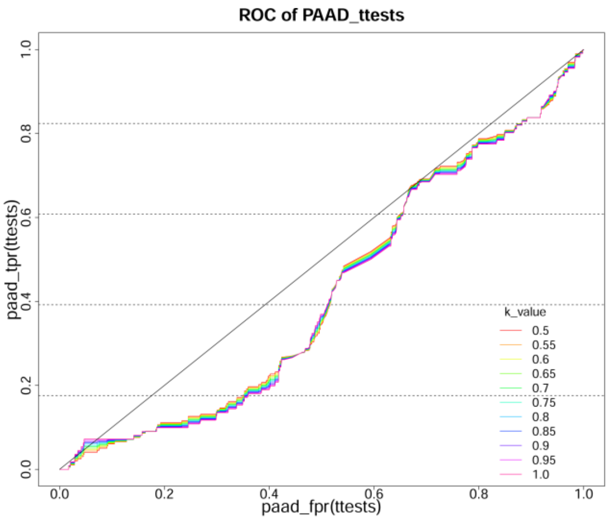
ROC of PAAD_ttests
paad_tpr(ttests)
paad_fpr(ttests)
0.0
0.2
0.4
0.6
0.8
1.0
k_value
0.5
0.55
0.6
0.65
0.7
0.75
0.8
0.85
0.9
0.95
1.0

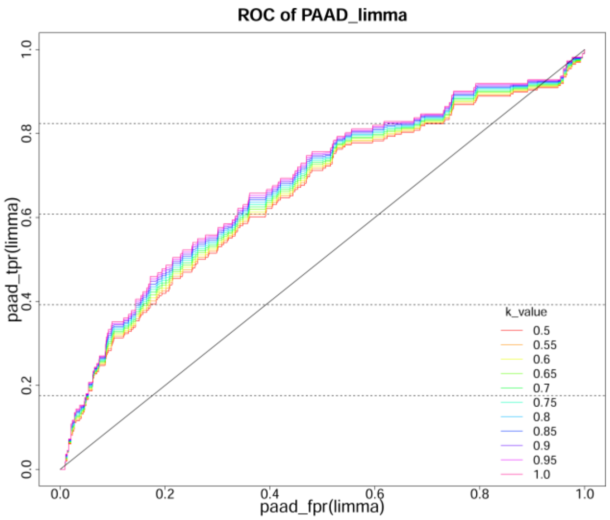
ROC of PAAD_limma
paad_tpr(limma)
paad_fpr(limma)
0.0
0.2
0.4
0.6
0.8
1.0
k_value
0.5
0.55
0.6
0.65
0.7
0.75
0.8
0.85
0.9
0.95
1.0

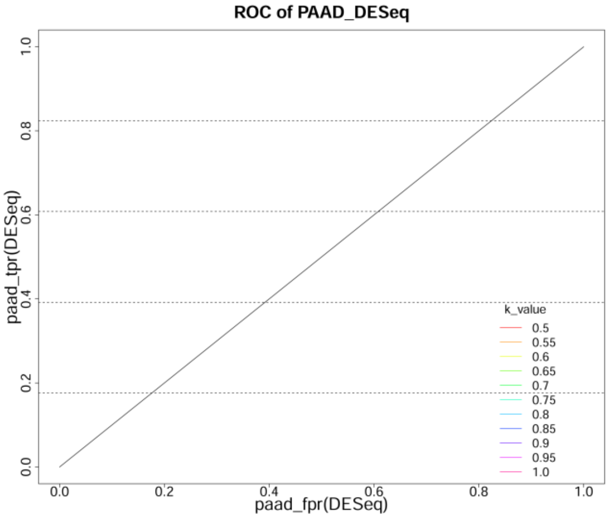
ROC of PAAD_DESeq
paad_tpr(DESeq)
paad_fpr(DESeq)
0.0
0.2
0.4
0.6
0.8
1.0
k_value
0.5
0.55
0.6
0.65
0.7
0.75
0.8
0.85
0.9
0.95
1.0

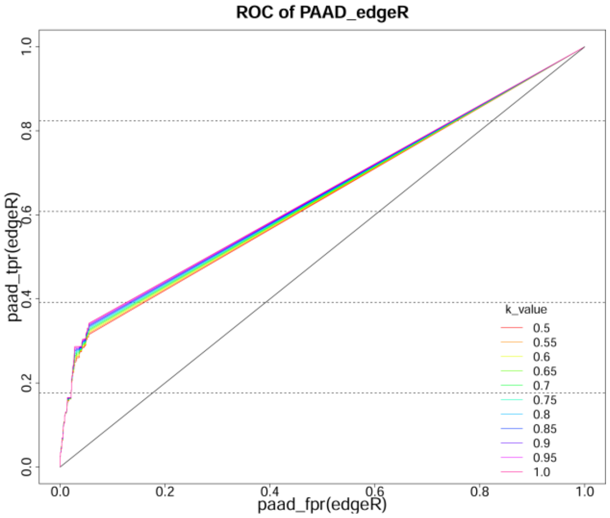
ROC of PAAD_edgeR
paad_tpr(edgeR)
paad_fpr(edgeR)
0.0
0.2
0.4
0.6
0.8
1.0
k_value
0.5
0.55
0.6
0.65
0.7
0.75
0.8
0.85
0.9
0.95
1.0

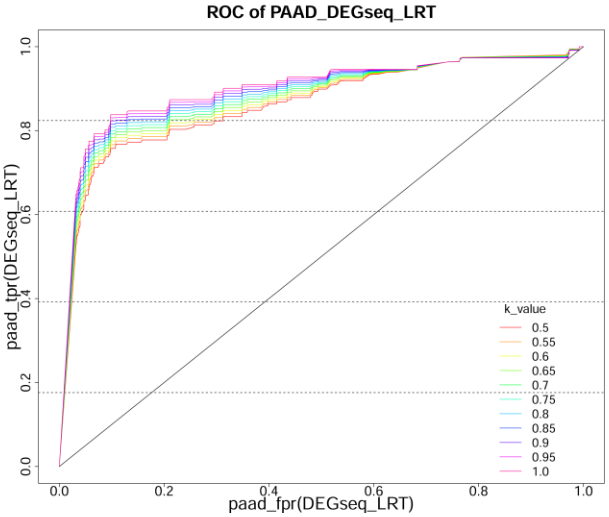
ROC of PAAD_DEGseq_LRT
paad_tpr(DEGseq_LRT)
paad_fpr(DEGseq_LRT)
0.0
0.2
0.4
0.6
0.8
1.0
k_value
0.5
0.55
0.6
0.65
0.7
0.75
0.8
0.85
0.9
0.95
1.0

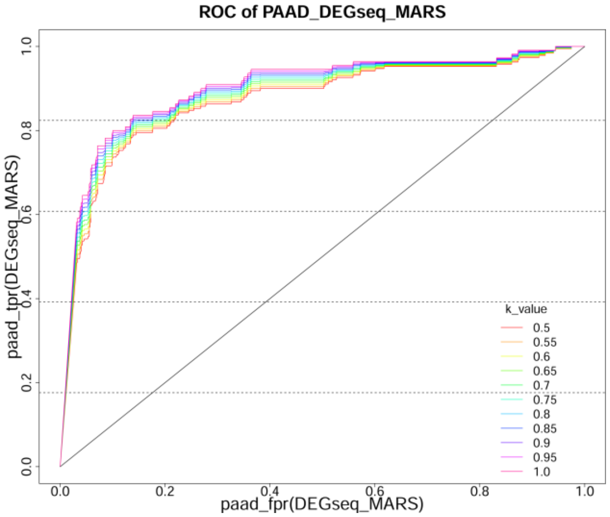
ROC of PAAD_DEGseq_MARS
paad_tpr(DEGseq_MARS)
paad_fpr(DEGseq_MARS)
0.0
0.2
0.4
0.6
0.8
1.0
k_value
0.5
0.55
0.6
0.65
0.7
0.75
0.8
0.85
0.9
0.95
1.0

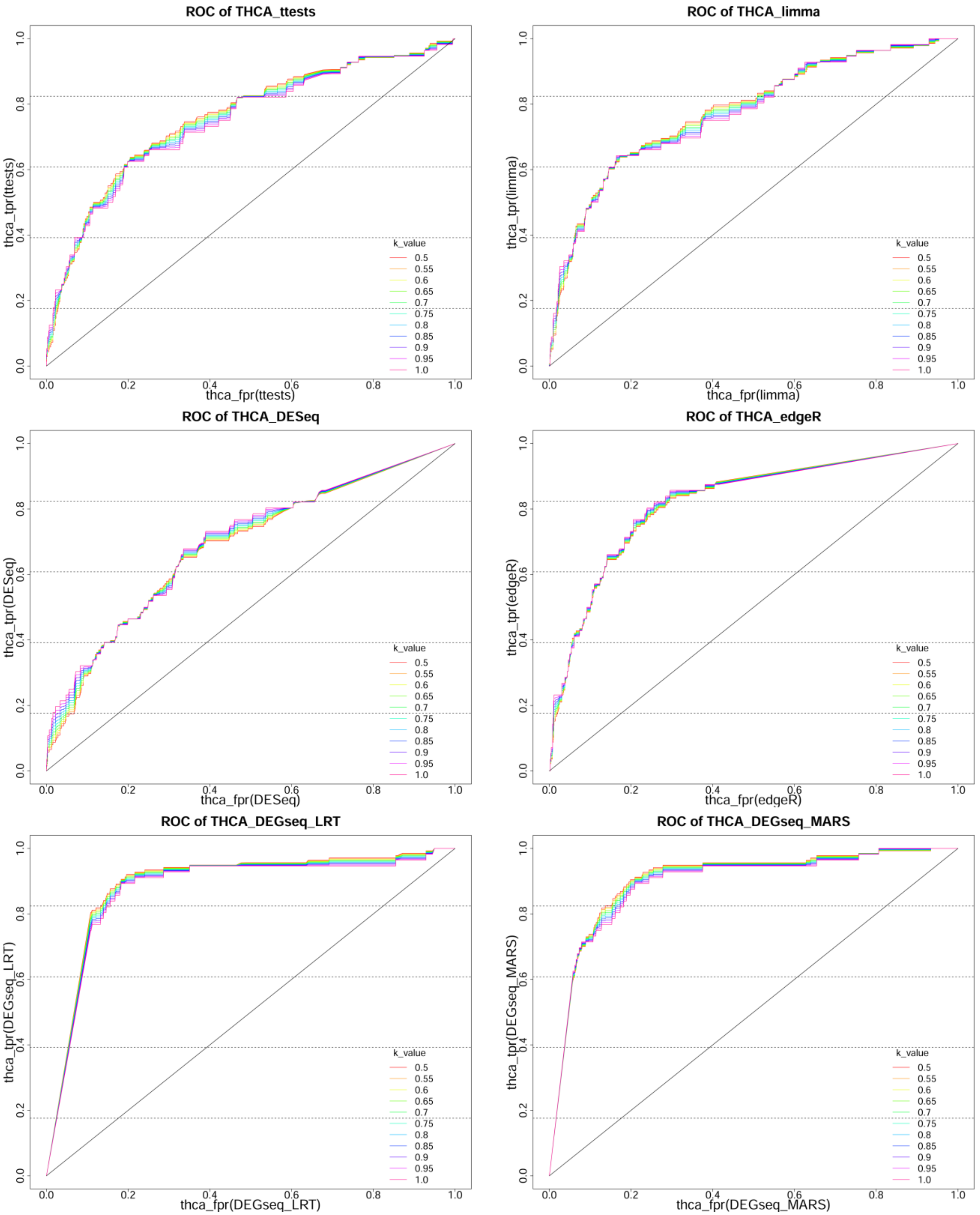

Figures S1-S4. The ROC of 6 methods on ESCA, LUAD, PAAD and THCA datasets based on integrated HMDD 2.0 and Infer microRNA-disease association.

These ROC are obtained from classification of miRNAs obtained from 6 methods (t-test, Limma, DESeq, edgeR, LRT and MARS) on 5 datasets based on the true class in integrated HMDD 2.0 and Infer microRNA-disease association and k-value is the weighting coefficient, which is arithmetic progression from 0.5-1 with the step size equaling to 0.05.

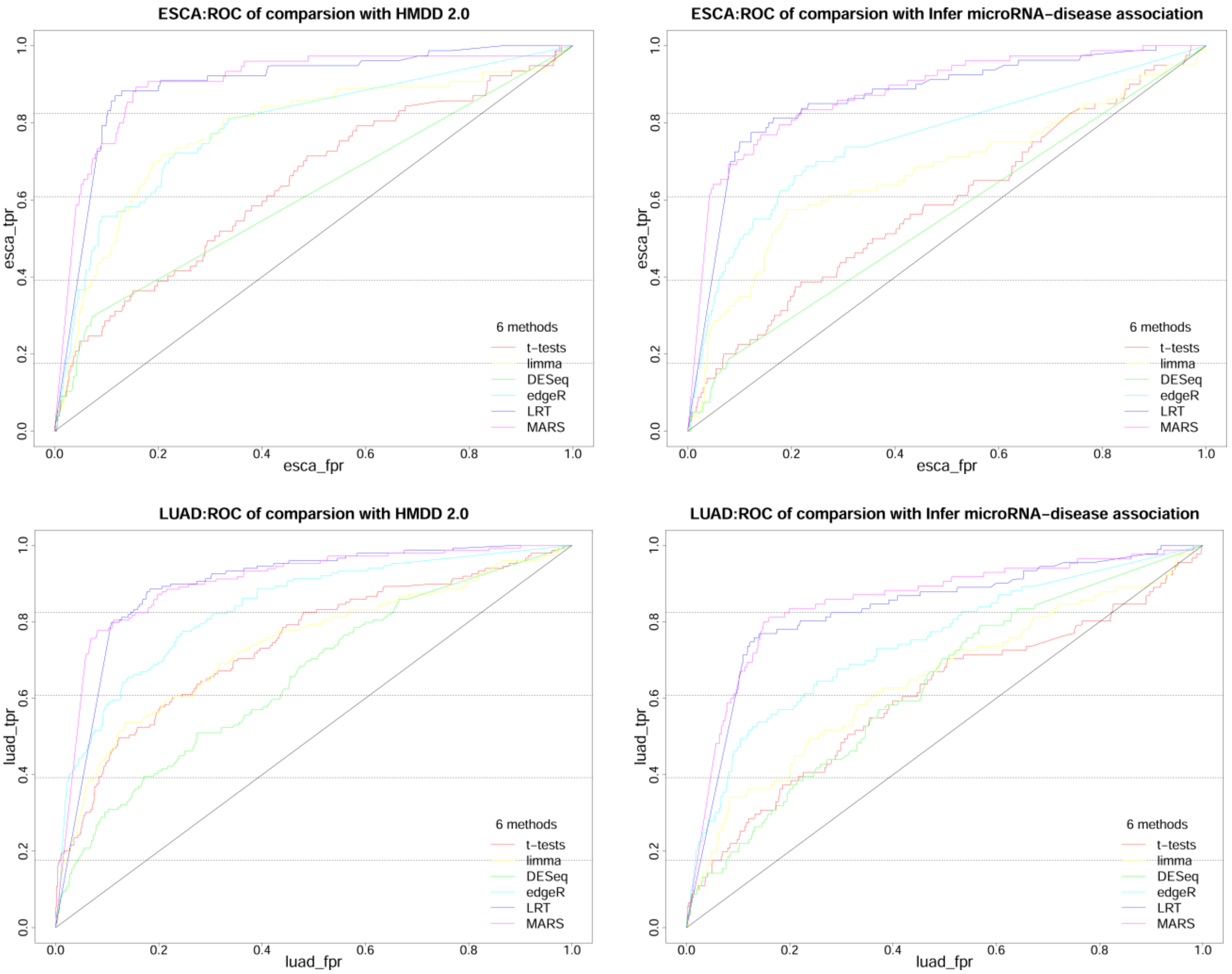

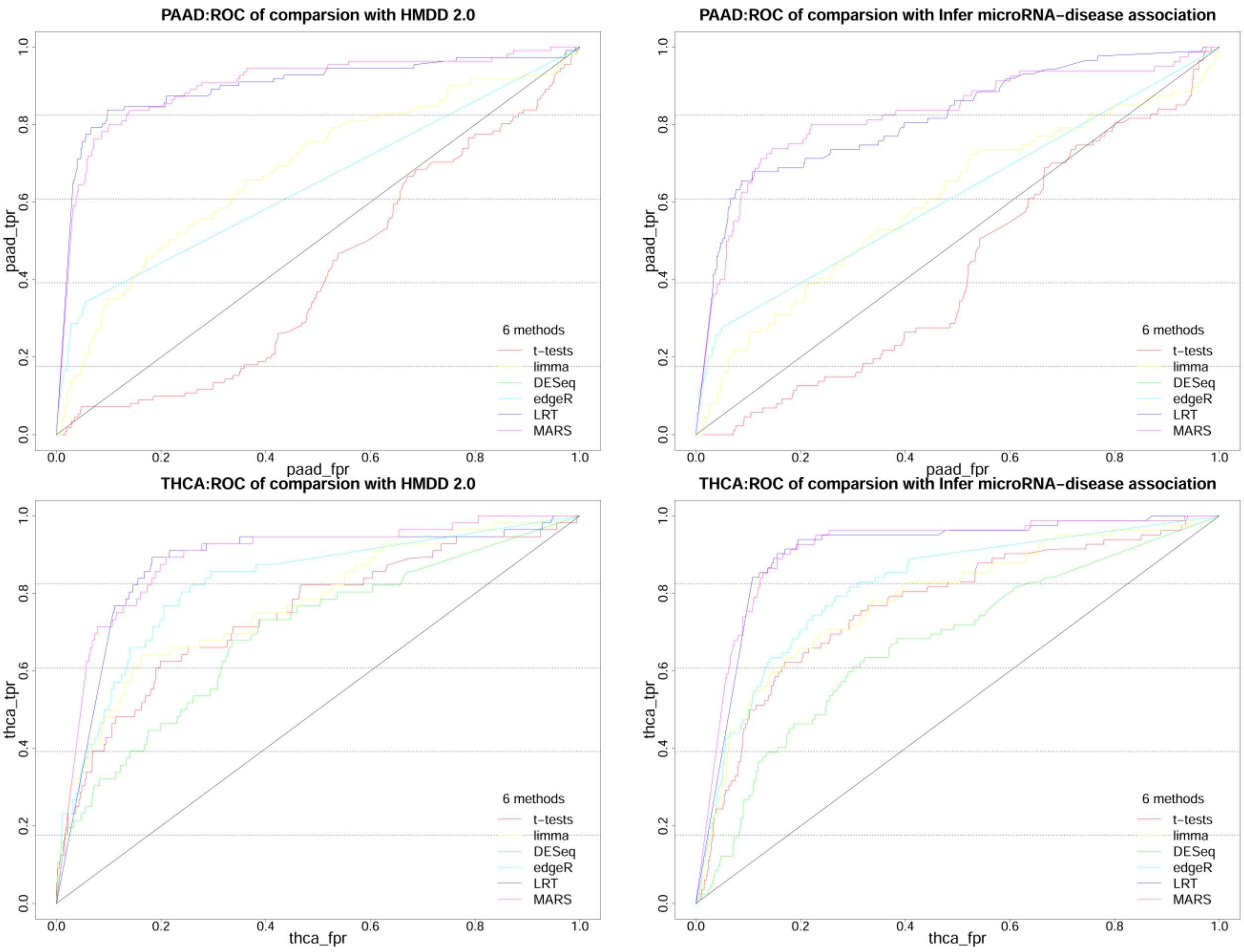

Figures S5-S8. The ROC of 6 methods on ESCA, LUAD, PAAD and THCA datasets based on independently HMDD 2.0 and Infer microRNA-disease association.

These ROC are obtained from classification of miRNAs obtained from 6 methods (t-test, Limma, DESeq, edgeR, LRT and MARS) on 5 datasets based on the true class in independently HMDD 2.0 and Infer microRNA-disease association.
