# Supplementary material for: Which statistical significance test best detects oncomiRNAs in cancer tissues? An exploratory analysis
Source: Oncotarget. 2016 Oct 23;7(51):85613–23. doi: 10.18632/oncotarget.12828 (PMC5356763; doi:10.18632/oncotarget.12828)
Supplement: Supplementary file 2 [file oncotarget-07-85613-s002.docx]

Which statistical significance test best detects oncomiRNAs in cancer tissues? An exploratory analysis

**Table S1.** The AUC of Figure S1-S5 (6 methods in 5 datasets in different k-values)

|  | AUC(k=0.5) | AUC(k=0.55) | AUC(k=0.60) | AUC(k=0.65) | AUC(k=0.70) | AUC(k=0.75) | AUC(k=0.80) | AUC(k=0.85) | AUC(k=0.90) | AUC(k=0.90) | AUC(k=1.00) |
| --- | --- | --- | --- | --- | --- | --- | --- | --- | --- | --- | --- |
| BRCA_ttests | 0.6221 | 0.6225 | 0.6228 | 0.6231 | 0.6234 | 0.6236 | 0.6238 | 0.6241 | 0.6243 | 0.6245 | 0.6246 |
| BRCA_limma | 0.7207 | 0.7179 | 0.7153 | 0.7129 | 0.7107 | 0.7087 | 0.7068 | 0.7050 | 0.7033 | 0.7018 | 0.7003 |
| BRCA_DESeq | 0.6159 | 0.6129 | 0.6103 | 0.6078 | 0.6055 | 0.6034 | 0.6014 | 0.5995 | 0.5978 | 0.5962 | 0.5947 |
| BRCA_edgeR | 0.7477 | 0.7470 | 0.7463 | 0.7457 | 0.7452 | 0.7447 | 0.7442 | 0.7438 | 0.7434 | 0.7430 | 0.7426 |
| BRCA_LRT | 0.8281 | 0.8286 | 0.8291 | 0.8296 | 0.8300 | 0.8305 | 0.8308 | 0.8312 | 0.8315 | 0.8318 | 0.8321 |
| BRCA_MARS | 0.8377 | 0.8393 | 0.8407 | 0.8421 | 0.8433 | 0.8444 | 0.8455 | 0.8465 | 0.8475 | 0.8483 | 0.8492 |
|  |  |  |  |  |  |  |  |  |  |  |  |
| ESCA_ttests | 0.6238 | 0.6251 | 0.6265 | 0.6280 | 0.6296 | 0.6313 | 0.6332 | 0.6353 | 0.6376 | 0.6400 | 0.6428 |
| ESCA_limma | 0.7411 | 0.7437 | 0.7465 | 0.7495 | 0.7528 | 0.7564 | 0.7603 | 0.7646 | 0.7694 | 0.7746 | 0.7805 |
| ESCA_DESeq | 0.5830 | 0.5859 | 0.5888 | 0.5918 | 0.5948 | 0.5977 | 0.6007 | 0.6038 | 0.6068 | 0.6098 | 0.6129 |
| ESCA_edgeR | 0.7747 | 0.7764 | 0.7780 | 0.7796 | 0.7813 | 0.7829 | 0.7846 | 0.7862 | 0.7879 | 0.7895 | 0.7912 |
| ESCA_LRT | 0.8813 | 0.8829 | 0.8846 | 0.8862 | 0.8878 | 0.8895 | 0.8911 | 0.8928 | 0.8945 | 0.8962 | 0.8978 |
| ESCA_MARS | 0.8913 | 0.8926 | 0.8938 | 0.8951 | 0.8964 | 0.8977 | 0.8990 | 0.9003 | 0.9016 | 0.9029 | 0.9043 |
|  |  |  |  |  |  |  |  |  |  |  |  |
| LUAD_ttests | 0.6891 | 0.6955 | 0.7016 | 0.7074 | 0.7130 | 0.7184 | 0.7235 | 0.7284 | 0.7331 | 0.7376 | 0.7419 |
| LUAD_limma | 0.7034 | 0.7075 | 0.7115 | 0.7153 | 0.7189 | 0.7223 | 0.7256 | 0.7288 | 0.7319 | 0.7348 | 0.7376 |
| LUAD_DESeq | 0.6468 | 0.6478 | 0.6488 | 0.6497 | 0.6506 | 0.6515 | 0.6523 | 0.6531 | 0.6538 | 0.6545 | 0.6552 |
| LUAD_edgeR | 0.8024 | 0.8066 | 0.8106 | 0.8144 | 0.8181 | 0.8216 | 0.8250 | 0.8282 | 0.8313 | 0.8343 | 0.8371 |
| LUAD_LRT | 0.8694 | 0.8722 | 0.8749 | 0.8774 | 0.8799 | 0.8822 | 0.8845 | 0.8866 | 0.8887 | 0.8907 | 0.8926 |
| LUAD_MARS | 0.8833 | 0.8856 | 0.8878 | 0.8898 | 0.8918 | 0.8937 | 0.8955 | 0.8972 | 0.8988 | 0.9004 | 0.9019 |
|  |  |  |  |  |  |  |  |  |  |  |  |
| PAAD_ttests | 0.4216 | 0.4209 | 0.4202 | 0.4196 | 0.4189 | 0.4183 | 0.4177 | 0.4171 | 0.4165 | 0.4160 | 0.4154 |
| PAAD_limma | 0.6475 | 0.6514 | 0.6553 | 0.6590 | 0.6626 | 0.6662 | 0.6696 | 0.6730 | 0.6763 | 0.6796 | 0.6827 |
| PAAD_DESeq | NA | NA | NA | NA | NA | NA | NA | NA | NA | NA | NA |
| PAAD_edgeR | 0.6330 | 0.6345 | 0.6360 | 0.6374 | 0.6388 | 0.6401 | 0.6414 | 0.6427 | 0.6440 | 0.6452 | 0.6464 |
| PAAD_LRT | 0.8628 | 0.8666 | 0.8704 | 0.8741 | 0.8777 | 0.8813 | 0.8847 | 0.8881 | 0.8914 | 0.8946 | 0.8978 |
| PAAD_MARS | 0.8690 | 0.8727 | 0.8763 | 0.8797 | 0.8831 | 0.8863 | 0.8895 | 0.8925 | 0.8955 | 0.8984 | 0.9012 |
|  |  |  |  |  |  |  |  |  |  |  |  |
| THCA_ttests | 0.7598 | 0.7586 | 0.7574 | 0.7561 | 0.7548 | 0.7533 | 0.7519 | 0.7504 | 0.7488 | 0.7471 | 0.7453 |
| THCA_limma | 0.7795 | 0.7789 | 0.7783 | 0.7777 | 0.7770 | 0.7763 | 0.7756 | 0.7748 | 0.7740 | 0.7732 | 0.7723 |
| THCA_DESeq | 0.6817 | 0.6829 | 0.6842 | 0.6855 | 0.6869 | 0.6884 | 0.6899 | 0.6914 | 0.6931 | 0.6948 | 0.6966 |
| THCA_edgeR | 0.8206 | 0.8208 | 0.8211 | 0.8214 | 0.8218 | 0.8221 | 0.8225 | 0.8228 | 0.8232 | 0.8236 | 0.8240 |
| THCA_LRT | 0.8914 | 0.8900 | 0.8885 | 0.8869 | 0.8852 | 0.8835 | 0.8817 | 0.8798 | 0.8779 | 0.8758 | 0.8737 |
| THCA_MARS | 0.9027 | 0.9017 | 0.9007 | 0.8997 | 0.8986 | 0.8975 | 0.8963 | 0.8951 | 0.8938 | 0.8925 | 0.8911 |

***Note:***

These AUC are from ROC obtained from classification of miRNAs obtained from 6 methods(T-test, Limma, DESeq, edgeR, LRT, MARS) on 5 datasets based on the true class in integrated HMDD 2.0 and Infer microRNA-disease association and k-value is the weighting coefficient, which is arithmetic progression from 0.5-1 with the step size equaling to 0.05.

**Table S1.** The AUC of Figure S1-S5 (6 methods in 5 datasets)

|  | AUC(HMDD 2.0) | AUC(Infer microRNA-disease association) |
| --- | --- | --- |
| BRCA_ttests | 0.6246 | 0.6157 |
| BRCA_limma | 0.7003 | 0.7725 |
| BRCA_DESeq | 0.5947 | 0.6699 |
| BRCA_edgeR | 0.7426 | 0.7603 |
| BRCA_DEGseq_LRT | 0.8321 | 0.8177 |
| BRCA_DEGseq_MARS | 0.8492 | 0.8091 |
|  |  |  |
| ESCA_ttests | 0.6428 | 0.5873 |
| ESCA_limma | 0.7805 | 0.6725 |
| ESCA_DESeq | 0.6129 | 0.5545 |
| ESCA_edgeR | 0.7912 | 0.7584 |
| ESCA_DEGseq_LRT | 0.8978 | 0.8654 |
| ESCA_DEGseq_MARS | 0.9043 | 0.8788 |
|  |  |  |
| LUAD_ttests | 0.7419 | 0.6024 |
| LUAD_limma | 0.7376 | 0.6474 |
| LUAD_DESeq | 0.6552 | 0.6331 |
| LUAD_edgeR | 0.8371 | 0.7460 |
| LUAD_DEGseq_LRT | 0.8926 | 0.8314 |
| LUAD_DEGseq_MARS | 0.9019 | 0.8508 |
|  |  |  |
| PAAD_ttests | 0.4154 | 0.4295 |
| PAAD_limma | 0.6827 | 0.6026 |
| PAAD_DESeq | NA | NA |
| PAAD_edgeR | 0.6464 | 0.6158 |
| PAAD_DEGseq_LRT | 0.8978 | 0.8180 |
| PAAD_DEGseq_MARS | 0.9012 | 0.8248 |
|  |  |  |
| THCA_ttests | 0.7453 | 0.7697 |
| THCA_limma | 0.7723 | 0.7844 |
| THCA_DESeq | 0.6966 | 0.6715 |
| THCA_edgeR | 0.8240 | 0.8182 |
| THCA_DEGseq_LRT | 0.8737 | 0.9036 |
| THCA_DEGseq_MARS | 0.8911 | 0.9107 |

***Note:***

These AUC are from ROC obtained from classification of miRNAs obtained from 6 methods(t-test, Limma, DESeq, edgeR, LRT, MARS) on 5 datasets based on the true class in independently HMDD 2.0 and Infer microRNA-disease association
